# Cytosolic Ca^2+^ shifts as early markers of cytotoxicity

**DOI:** 10.1186/1478-811X-11-11

**Published:** 2013-02-06

**Authors:** Philippe Wyrsch, Christian Blenn, Theresa Pesch, Sascha Beneke, Felix R Althaus

**Affiliations:** 1 Institute of Pharmacology and Toxicology, University of Zurich-Vetsuisse, Winterthurerstrasse 260, Zurich, CH-8057, Switzerland

**Keywords:** Alamar blue, Arsenic trioxide, Fluo-4, Gossypol, H_2_O_2_, Staurosporine

## Abstract

The determination of the cytotoxic potential of new and so far unknown compounds as well as their metabolites is fundamental in risk assessment. A variety of strategic endpoints have been defined to describe toxin-cell interactions, leading to prediction of cell fate. They involve measurement of metabolic endpoints, bio-energetic parameters or morphological cell modifications. Here, we evaluated alterations of the free cytosolic Ca^2+^ homeostasis using the Fluo-4 dye and compared results with the metabolic cell viability assay Alamar Blue. We investigated a panel of toxins (As_2_O_3_, gossypol, H_2_O_2_, staurosporine, and titanium(IV)-salane complexes) in four different mammalian cell lines covering three different species (human, mouse, and African green monkey). All tested compounds induced an increase in free cytosolic Ca^2+^ within the first 5 s after toxin application. Cytosolic Ca^2+^ shifts occurred independently of the chemical structure in all tested cell systems and were persistent up to 3 h. The linear increase of free cytosolic Ca^2+^ within the first 5 s of drug treatment correlates with the EC_25_ and EC_75_ values obtained in Alamar Blue assays one day after toxin exposure. Moreover, a rise of cytosolic Ca^2+^ was detectable independent of induced cell death mode as assessed by caspase and poly(ADP-ribose) polymerase (PARP) activity in HeLa versus MCF-7 cells at very low concentrations. In conclusion, a cytotoxicity assay based on Ca^2+^ shifts has a low limit of detection (LOD), is less time consuming (at least 24 times faster) compared to the cell viability assay Alamar Blue and is suitable for high-troughput-screening (HTS).

## Background

The development of assays estimating the cytotoxic potential of drugs and chemicals is of fundamental interest in early risk assessment to prioritize them for further testing. Moreover, a few years ago, the European Union (EU) initiated a regulation for the Registration, Evaluation and Authorisation of Chemicals (REACH). Around 30 000 chemical substances, which are manufactured, imported or, used in the EU require validation [1,2]. The implementation of REACH will increase the demand of cytotoxicity testing and risk assessment.

In the past, a variety of different biological endpoints have been defined for cytotoxicity testing. These include the assessment of energy status (ATP depletion, ATP/ADP ratio), cell membrane integrity (Neutral red, Trypan blue, lactate dehydrogenase (LDH) leakage), DNA-strand breaks (COMET) as well as metabolic parameters (3-(4,5-Dimethylthiazol-2-yl)-2,5-diphenyltetrazolium bromide (MTT), Alamar Blue) [3-5]. The evaluation of these parameters is often time and cost intensive and several different endpoints must be considered for a final decision.

**Figure 1:**
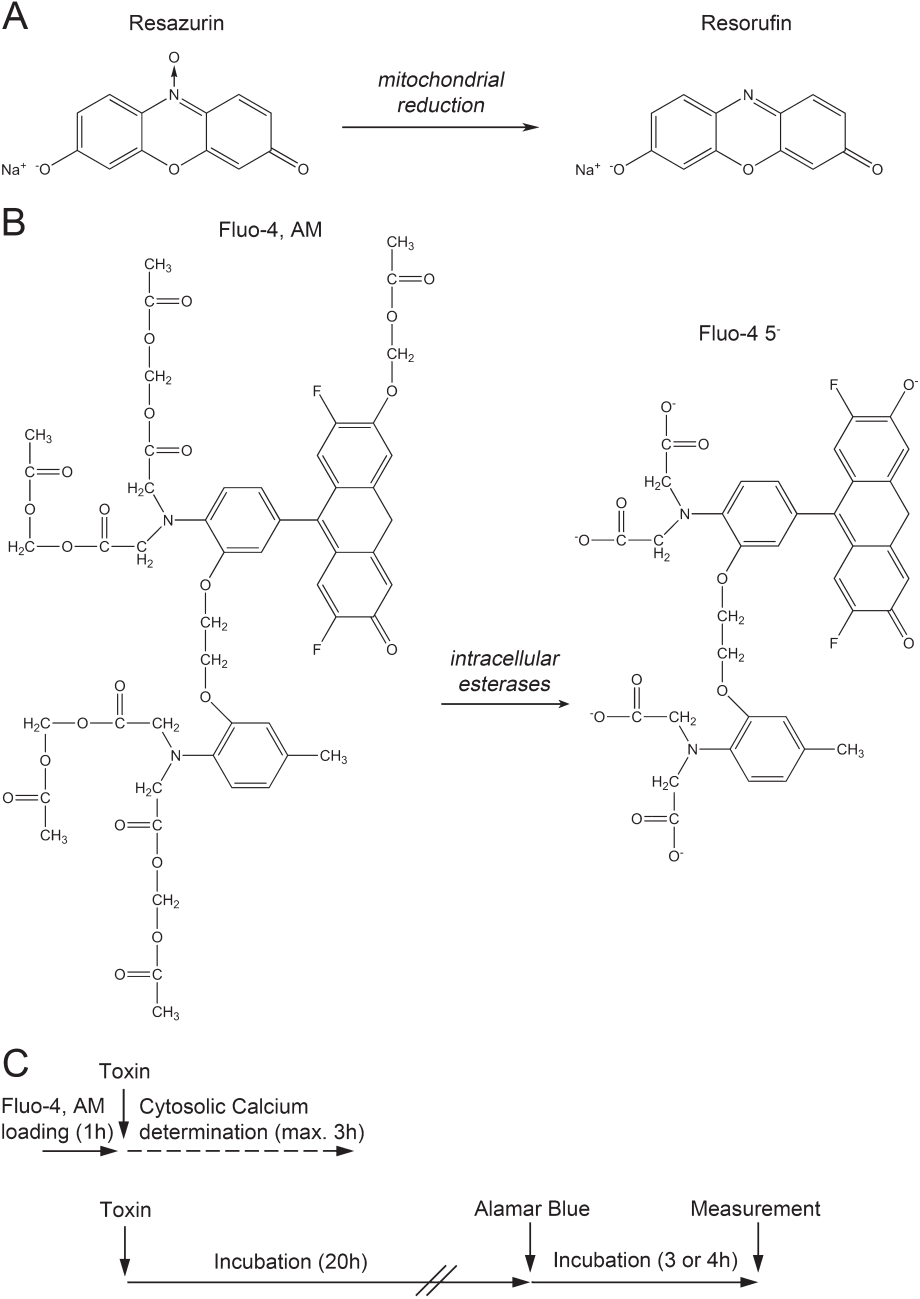
**Principle and experimental setup for cytotoxicity determination.** (**A**) Alamar Blue conversion (**B**) Principle of Fluo-4 assay for free cytosolic Ca^2+^ determination (**C**) Experimental time line for Fluo-4 and Alamar blue assays as investigated in this study.

**Figure 2:**
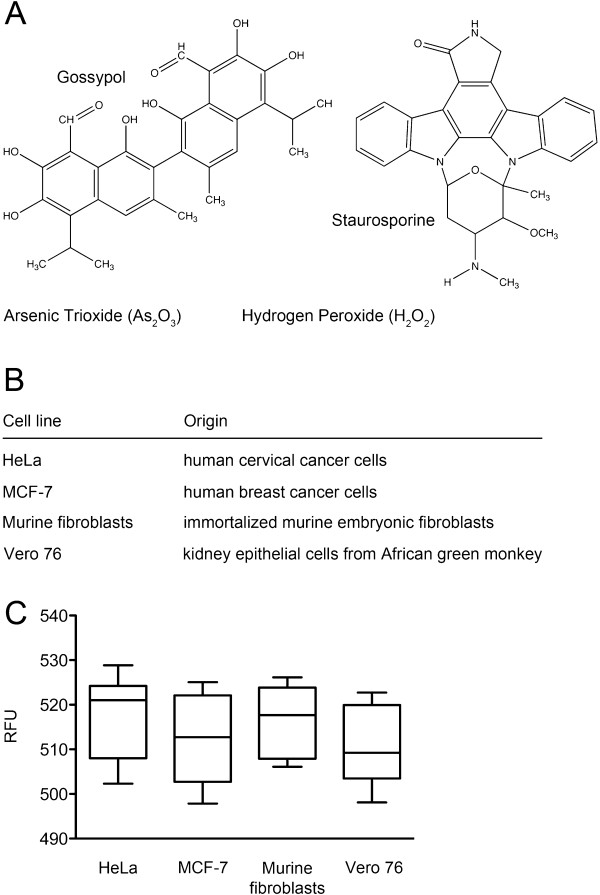
**Toxins, cells and Fluo-4 activation capacities.** (**A**) Chemical structures of the investigated compounds (**B**) List of mammalian cells analyzed in this study (**C**) HeLa, MCF-7, murine fibroblasts and Vero 76 cells were incubated with Fluo-4, AM as described in Methods and relative fluorescent (RFU) was determined (mean±SD; n=8).

The determination of metabolic activity using the Alamar Blue viability assay is based on mitochondrial hydrolase activity that is generally affected by many different drugs as well as radiation [6-8]. Blue resazurin is metabolized into pink resorufin by viable cells and this color change quantifies the amount of intact cells (Figure 1A). Here, we evaluated the toxicity of four model compounds in adherent cell cultures from three different species: human cervical (HeLa) and breast cancer cells (MCF-7), murine fibroblasts and kidney epithelial cells from African green monkey (Vero 76) (Figure 2A, B). We compared the cytotoxicity of arsenic trioxide (As_2_O_3_), gossypol, hydrogen peroxide (H_2_O_2_) and staurosporine in Alamar Blue assays with toxin-induced elevations of cytosolic Ca^2+^ (Figure 1C) measured by Fluo-4 (Figure 1B). The choice of these test compounds aims to cover a broad spectrum of different chemical structures and cytotoxicity mechanisms:

1. As_2_O_3_ cytotoxicity is characterized by activation of the caspase cascade, simultaneous stress kinase signaling, the generation of reactive oxygen species (ROS) oxidizing macromolecules, and a disturbed endoplasmic reticulum function [9-13]. However, the detailed mechanisms by which arsenic interferes with living cells are not fully understood.

2. The racematic organic compound gossypol isolated from cotton seed and its metabolites display a wide pattern of cytotoxic cell alterations because of the complexity of gossypol chemistry and its potential chemical reactions with other macromolecules. Gossypol cytotoxicity includes ROS induction, microsomal enzyme inhibition, glutathione-S-transferase inhibition, mitochondrial dysfunction, caspase dependent and independent cell death associated with DNA degradation, and was described to interfere with the anti-apoptotic bcl-2 protein [14-18].

3. In this study, H_2_O_2_ is used as surrogate for ROS. It oxidizes directly macromolecules including lipids, proteins and DNA. This can lead to a complex cytotoxicity response with the involvement of stress activated kinases, caspase and calpain activation, mitochondrial apoptosis induction factor (AIF) translocation, endoplasmic reticulum stress, nuclear poly(ADP-ribosylation), DNA degradation and many more [19-22].

4. The bacterial alkaloide staurosporine is intensively investigated as inducer of a classical apoptotic cell death. It was initially described as an inhibitor of protein kinases [23-25]. On cellular level it leads to interruption of mitochondrial membranes, resulting in cytochrome c efflux and, as a consequence, to caspase dependent cell death [26-28].

The Alamar Blue assay was considered as a benchmark cytotoxicity test because of its improved performance compared to other pertinent assays, e.g. detection of cell densities as low as 200 cells/well [29,30]. Moreover, the Alamar Blue viability assay is suitable for high-throughput-screening (HTS) to identify cytotoxic compounds regardless of the chemical class and the underlying mechanism.

Changes in free cytosolic Ca^2+^ were investigated using the fluorescent Ca^2+^ binding dye Fluo-4 during the application of four toxins in all cell lines (Figure 1B). Cellular calcium levels are tightly regulated in cells. Under physiological conditions the Ca^2+^ concentration in the cytosol is several magnitudes below the Ca^2+^ in the extracellular space (10^-7^ M versus 10^-3^ M, respectively [31]). Multiple cellular Ca^2+^ stores contribute to the maintenance of Ca^2+^ homeostasis and virtually all cell organelles control the transport of Ca^2+^ across their membranes to regulate organelle/cellular function [31]. It is well established that imbalances in cellular Ca^2+^ homeostasis can lead to a variety of different cell stress responses including the induction of cell death [32].

In our study, we focussed on the sensitivity, the species-specificity and the limit of detection (LOD) of the Fluo-4 Ca^2+^ assay. Sensitivity in our setting is defined as the ability to detect a significant effect of the used compounds at a specified concentration, whereas LOD is the lowest concentration level determined to be statistically different from blank. Here we show that As_2_O_3_, gossypol, H_2_O_2_ and staurosporine induce a dose-dependent increase in cytosolic Ca^2+^ at lethal (EC_75_) and sublethal (EC_25_) concentrations immediately after application in all tested cell lines. The cytosolic Ca^2+^ elevation follows linear kinetics for the first 5 s under all test conditions. Cytosolic Ca^2+^ shifts occur independent of the chemical structure of the toxin in all tested cell systems and are persistent up to 3 h. Moreover, the increase of free cytosolic Ca^2+^ is detectable independent of the mode of cell death as investigated by caspase and PARP activity. Therefore, we suggest the determination of early cytosolic Ca^2+^ shifts as a rapid, highly efficient, inexpensive cytotoxicity test that is at least as sensitive as the established metabolic assay Alamar Blue.

## Results

### The Ca^2+^ sensitive marker Fluo-4 is equally bio-activated in human, murine and monkey cells

Cytosolic Ca^2+^ was assessed using the fluorescence dye Fluo-4 (Figure 1B,C). This displays a high affinity to complex with Ca^2+^ ions (*K*_D_ of 345 nM) after its intracellular bio-activation by esterases [33]. Therefore, we first investigated the background fluorescence without any cytotoxic stress in HeLa, MCF-7, murine fibroblasts and Vero 76 cells to exclude any cell specific differences of Fluo-4, AM uptake and metabolism. We detected no differences between the tested cell lines under standard experiment conditions (Figure 2C).

### The EC_25_ and EC_75_ values of As_2_O_3_, gossypol, H_2_O_2_ and staurosporine assessed in Alamar blue assays correlate with immediate cytosolic Ca^2+^ rises in HeLa cells

We investigated the cytotoxic potential of the four toxins of interest in Alamar Blue viability assays as described in Methods (Figure 1A,C) and tested afterwards lethal and sublethal concentrations against changes in cytosolic Ca^2+^ homeostasis. The cytosolic Ca^2+^ levels remained unaffected for the whole measuring period in the absence of a toxic insult (Additional file 1A).

**Figure 3:**
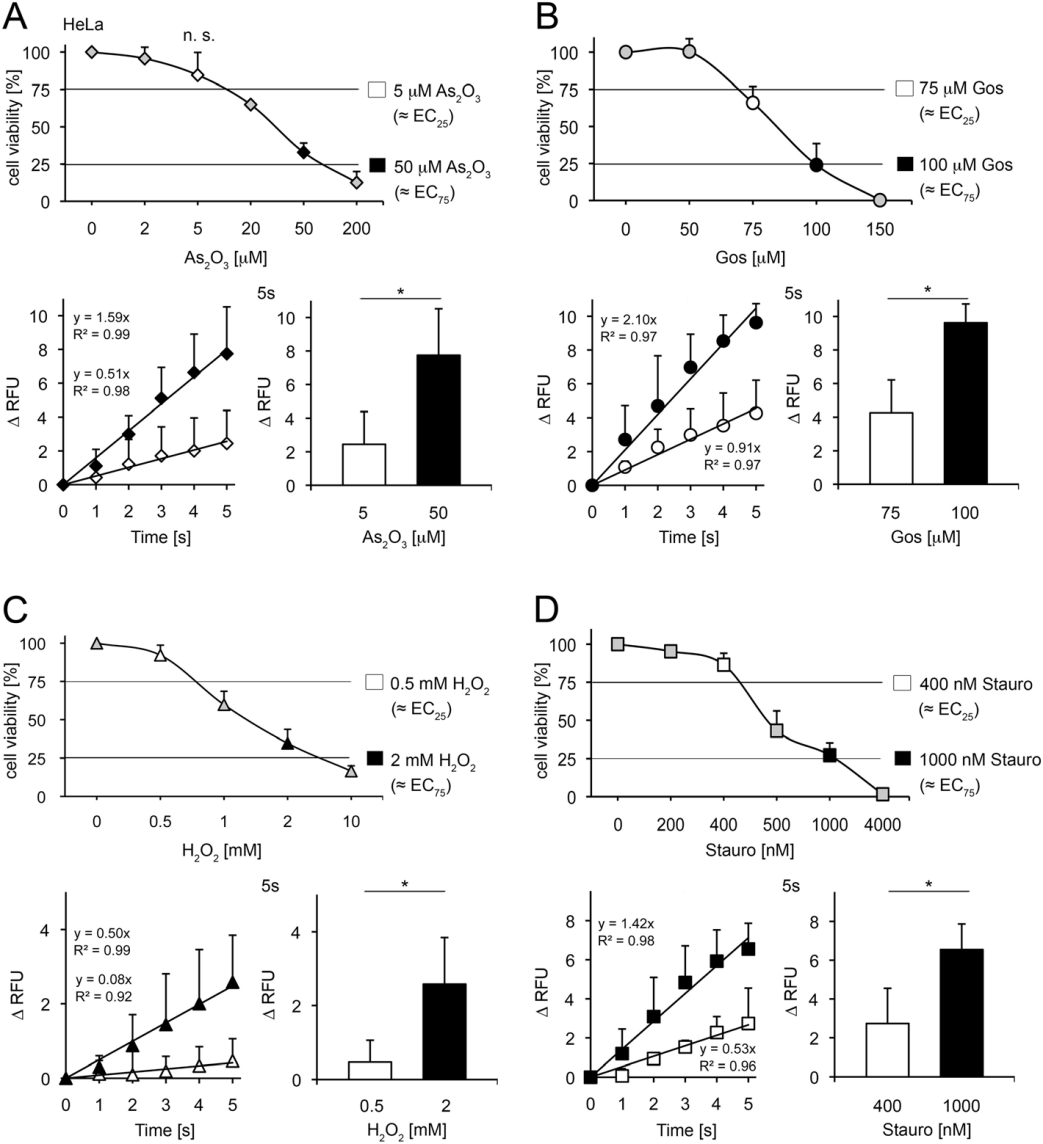
**Assessment of As**_**2**_**O**_**3**_**, gossypol, H**_**2**_**O**_**2 **_**and staurosporine-induced toxicity in HeLa cells.** (**A**) Upper panel: Alamar Blue assay in presence of As_2_O_3_ as indicated (mean±SD; n≥3; n.s. not significant; *t* test). Lower panel: Fluo-4 analysis of 5 μM and 50 μM As_2_O_3_ treated cells (mean±SD; *p<0.025; n=3; *t* test) (**B**) Upper panel: Alamar Blue assay in presence of gossypol as indicated (mean±SD; n≥3). Lower panel: Fluo-4 analysis of 75 μm and 100 μM gossypol treated cells (mean±SD; *p<0.0025; n≥4; *t* test) (**C**) Upper panel: Alamar Blue assay in presence of H_2_O_2_ as indicated (mean±SD; n≥4). Lower panel: Fluo-4 analysis of 0.5 mM and 2 mM H_2_O_2_ treated cells (mean±SD; *p<0.025; n≥3; *t* test) (**D**) Upper panel: Alamar Blue assay in presence of staurosporine as indicated (mean±SD; n≥3). Lower panel: Fluo-4 analysis of 400 nM and 1000 nM staurosporine treated cells (mean±SD; *p<0.05; n=3; *t* test).

As_2_O_3_ reduced the cell viability of HeLa cells dose dependently in Alamar Blue assays (Figure 3A). EC_25_ and EC_75_ values of 5 and 50 μM were obtained, respectively. These concentrations were analyzed in Fluo-4 assays. Indeed, As_2_O_3_ provoked a cytosolic Ca^2+^ increase that was persistent until the end of the measurement (1800 s, Additional file 2A) in a dose-dependent fashion. Cytosolic Ca^2+^ rose immediately after As_2_O_3_ application and followed linear kinetics within the first 5 s (Figure 3A). The cytosolic Ca^2+^ shifts differed significantly between 5 and 50 μM As_2_O_3_ already at this early time point (2.4±1.94 RFU versus 7.7±2.78 RFU; Figure 3A). The differences in cytosolic Ca^2+^ increases reflect the cytotoxicity values in Alamar Blue assays one day after toxin challenge, but already after 5 s.

Next, racematic gossypol was tested in Alamar Blue assays and compared with Fluo-4 analyses. Alamar Blue EC_25_ (75 μM) as well as EC_75_ (100 μM) induced cytosolic Ca^2+^ shifts in HeLa cells (Figure 3B, Additional file 2B). The increase of cytosolic Ca^2+^ signals was consistent for the whole period of observation (1800 s; 95.3±9.54 RFU versus 134.3±4.24 RFU, Additional file 2B). Interestingly, the Ca^2+^ increases followed linear kinetics within the first 5 s after treatment and manifested dose dependent differences at this early time point (Figure 3B).

Similar results were obtained when HeLa cells were challenged with oxidative stress inducer H_2_O_2_ (Figure 3C, Additional file 2C). 0.5 mM (EC_25_) and 2 mM (EC_75_) of H_2_O_2_ were analyzed regarding cytosolic Ca^2+^ imbalances. A dose dependency in the cytosolic Ca^2+^ response was already significant within the first 5 s of measurements (Figure 3C) and it was maintained until the end of the experiments (Additional file 2C).

Staurosporine toxicity was analyzed in a similar way (Figure 3D, Additional file 2D). Again, 400 nM (EC_25_) and 1 μM (EC_75_) determined in Alamar Blue assays correlate with linear increases in cytosolic Ca^2+^ levels for the first 5 s of Fluo-4 measurements (Figure 3D). In a next step, HeLa cells were challenged with doses below the EC_25_ of the corresponding toxin. There were no differences detectable between the control and the As_2_O_3_, gossypol and staurosporine treated cells after 5 s (Additional file 1E). These results are identical to the data obtained with Alamar Blue assay after 24 h. Again, no significant difference was measured comparing the control cells with the As_2_O_3_, gossypol and staurosporine treated cells (Additional file 1F).

Additionally, we compared two structurally highly related titanium(IV)-salane complexes (Additional file 1G) for their toxicity in HeLa cells. As described earlier, both showed expected behaviour in Alamar Blue assay, i.e. cytotoxicity of TC52 and no impact on viability by TC53 [34]. These findings were reproduced in our assay, with enhanced cytosolic Ca^2+^ fluxes at EC_25_ and EC_75_ in case of TC52, and no significant variation of cytosolic Ca^2+^ levels by TC53 (Additional file 1H,I).

In a next set of experiments we tested the hypothesis that prolonged incubation with an established calcium channel activator can also promote cell death due to an overload in free cytosolic Ca^2+^ (Additional file 3). Hela cells express purinergic P2X transmembranous Ca^2+^ channels and a known ligand for this type of plasma membrane channels is ATP, but only when applied in the extracellular environment [35-38]. The toxicity of extracellular ATP is well established in a variety of cell types and was shown to be mediated by especially P2X_7_ activation in HeLa cells [35,39-45]. Therefore we investigated the toxicity of ATP in this cell type and found that the EC_25_ as well as the EC_75_ deduced from Alamar blue assays (Additional file 3A) are reflected in dose dependent elevations of free cytosolic Ca^2+^ when assessed with the Fluo-4 dye (Additional file 3B). Again, this continuous over activation of P2X and possibly others related channels due to the specific ligand ATP results in a linear increase in the Fluo-4 signal within the first 5 s of treatment (Additional file 3C).

### Early changes of cytosolic Ca^2+^ accompany As_2_O_3_, gossypol, H_2_O_2_ and staurosporine induced toxicity in MCF-7 cells

**Figure 4:**
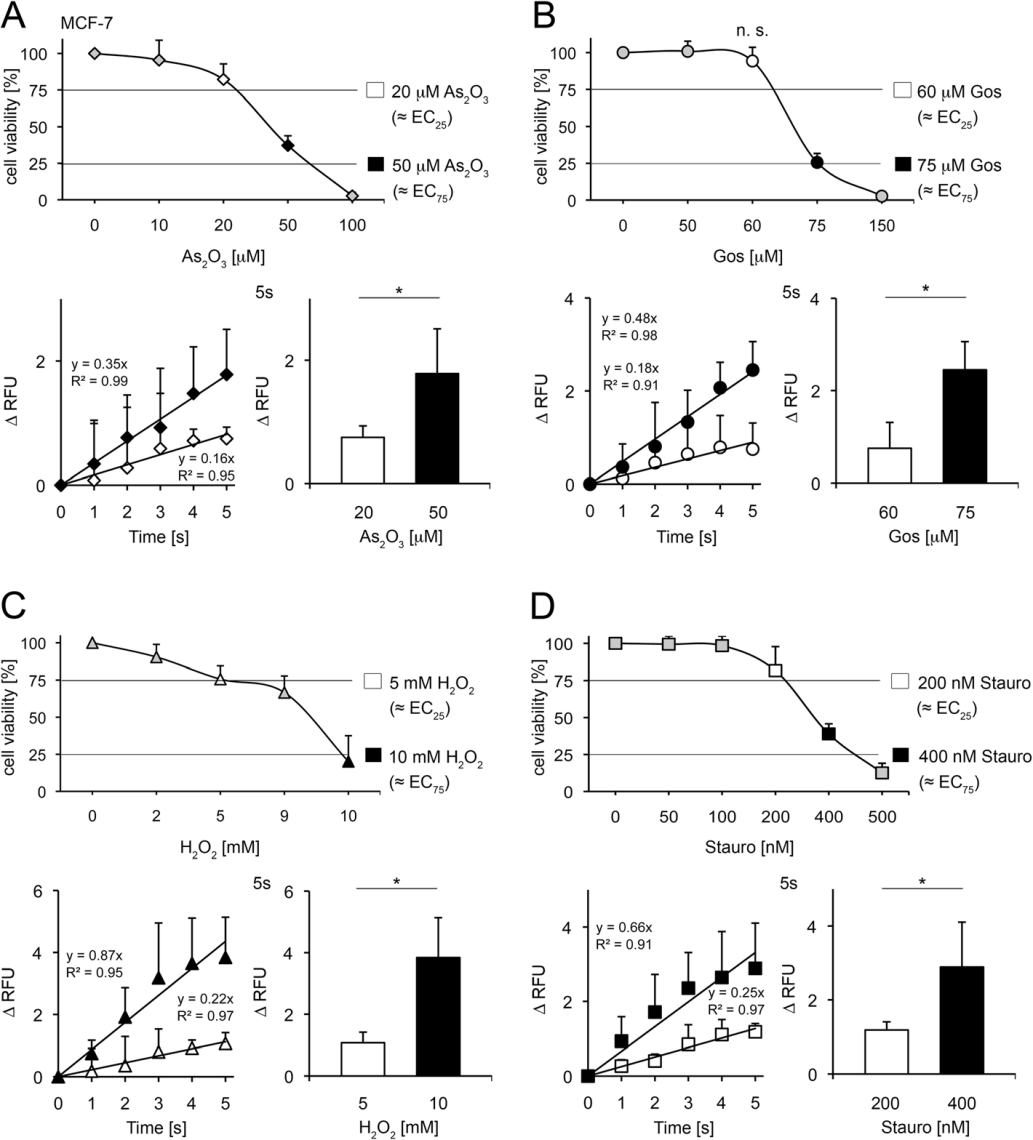
**Assessment of As**_**2**_**O**_**3**_**, gossypol, H**_**2**_**O**_**2 **_**and staurosporine-induced toxicity in MCF-7 cells.** (**A**) Upper panel: Alamar Blue assay in presence of As_2_O_3_ as indicated (mean±SD; n≥4). Lower panel: Fluo-4 analysis of 20 μM and 50 μM As_2_O_3_ treated cells (mean±SD; *p<0.05; n=3; *t* test) (**B**) Upper panel: Alamar Blue assay in presence of gossypol as indicated (mean±SD; n≥4; n.s. not significant; *t* test). Lower panel: Fluo-4 analysis of 60 μm and 75 μM gossypol treated cells (mean±SD; *p<0.025; n=4; *t* test) (**C**) Upper panel: Alamar Blue assay in presence of H_2_O_2_ as indicated (mean±SD; n≥4). Lower panel: Fluo-4 analysis of 5 mM and 10 mM H_2_O_2_ treated cells (mean±SD; *p<0.005; n≥3; *t* test) (**D**) Upper panel: Alamar Blue assay in presence of staurosporine as indicated (mean±SD; n≥4). Lower panel: Fluo-4 analysis of 200 nM and 400 nM staurosporine treated cells (mean±SD; *p<0.05; n≥3; *t* test).

We analyzed the toxicity of the four test compounds in the second human cell line MCF-7 (Figure 4A-D, Additional file 4A-D). The EC_25_ and EC_75_ concentrations of all toxins (20 μM and 50 μM As_2_O_3_, 60 μM and 75 μM gossypol, 5 mM and 10 mM H_2_O_2_, 0.2 μM and 0.4 μM staurosporine) were analyzed in Fluo-4 assays directly after application. Cytoplasmic Ca^2+^ was not altered in untreated control MCF-7 cells within 2 h (Additional file 1B). All toxins generated a dose dependent increase in cytosolic Ca^2+^ with linear kinetics within the first 5 s of the measurements (Figure 4A-D). Values at the EC_25_ and EC_75_ doses varied significantly not only at time point 5 s, but also for at least 30 min after the toxin treatment for all tested drugs (Additional file 4A-D).

### Drug-dependent elevations of cytosolic Ca^2+^ indicate As_2_O_3_, gossypol, H_2_O_2_ and staurosporine cytotoxicity in murine fibroblasts

**Figure 5:**
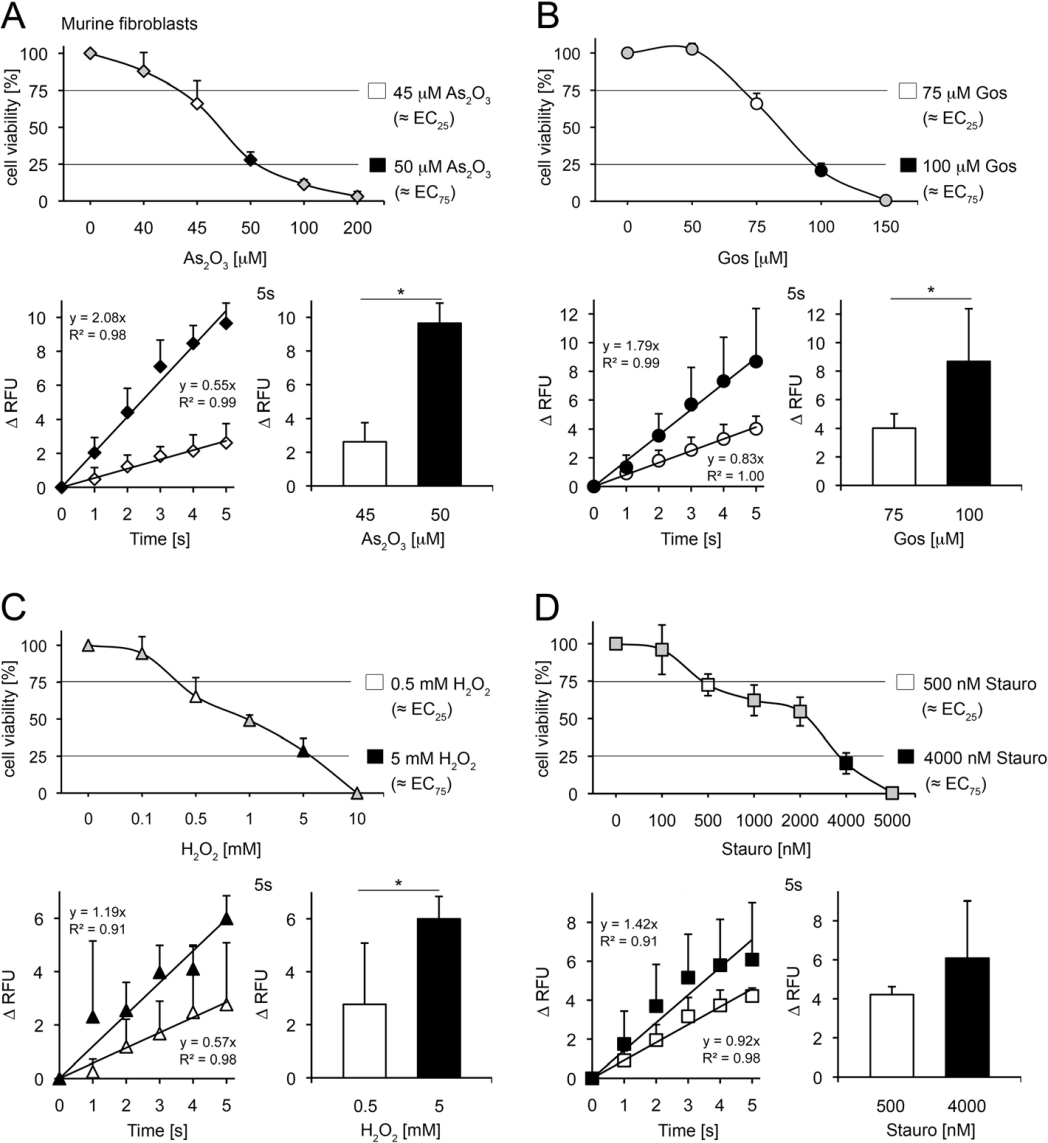
**Assessment of As**_**2**_**O**_**3**_**, gossypol, H**_**2**_**O**_**2 **_**and staurosporine-induced toxicity in murine fibroblasts.** (**A**) Upper panel: Cells were treated with increasing concentrations of As_2_O_3_ as indicated and afterwards cell viability was analysed with Alamar Blue (mean±SD; n≥4). Lower panel: Fluo-4 analysis of 45 μM and 50 μM As_2_O_3_ treated cells (mean±SD; *p<0.0001; n≥4; *t* test) (**B**) Upper panel: Cells were treated with increasing concentrations of gossypol as indicated and afterwards cell viability was analysed with Alamar Blue (mean±SD; n≥4). Lower panel: Fluo-4 analysis of 75 μM and 100 μM gossypol treated cells (mean±SD; *p<0.05; n=4; *t* test) (**C**) Upper panel: Alamar Blue assay in presence of H_2_O_2_ as indicated (mean±SD; n≥3). Lower panel: Fluo-4 analysis of 0.5 mM and 5 mM H_2_O_2_ treated cells (mean±SD; *p<0.01; n≥3; *t* test) (**D**) Upper panel: Alamar Blue assay in presence of staurosporine as indicated (mean±SD; n≥3). Lower panel: Fluo-4 analysis of 500 nM and 4000 nM staurosporine treated cells (mean±SD; n. s. not significant; n≥3; *t* test).

In the next set of experiments, the cytotoxicity of the drugs in murine fibroblasts was examined (Figure 5A-D, Additional file 5A-D). As expected, untreated control fibroblasts did not show any alteration in free cytosolic Ca^2+^ levels (Additional file 1C).

Whereas 45 μM As_2_O_3_ killed around 25% of murine fibroblasts, 50 μM represents the EC_75_ value in the Alamar Blue assay one day after drug exposure. By using these concentrations in Fluo-4 assays, a linear increase of cytosolic Ca^2+^ within the first 5 s in the presence of As_2_O_3_ was detected (2.6±1.14 RFU versus 9.6±1.20 RFU). The cytoplasmic Ca^2+^ slopes of the tested toxin concentrations were dose dependent and the RFUs at 5 s (Figure 5A) and 3 min (Additional file 5A) differed significantly between sublethal and lethal amounts of As_2_O_3_.

Gossypol (75 μM and 100 μM), H_2_O_2_ (0.5 mM and 5 mM) and staurosporine (0.5 μM and 4 μM) – concentration indicative of sublethal and lethal cell stress – were analysed in a similar way (Figure 5B-D, Additional file 5B-D). All these toxins confirmed a functional relationship between the applied dose and immediate alteration in cytoplasmic Ca^2+^ homeostasis. Moreover, the dose dependent differences in Fluo-4 determinations lasted up to 30 min post treatment (Additional file 5B,C). However, despite a significant rise in cytosolic Ca^2+^ level compared to control values at all time points tested, the observed increase between EC_25_ and EC_75_ was not statistically different after staurosporine treatment (Figure 5D and Additional file 5D).

### Determination of As_2_O_3_, gossypol, H_2_O_2_ and staurosporine mediated cytotoxicity in Vero 76 cells

**Figure 6:**
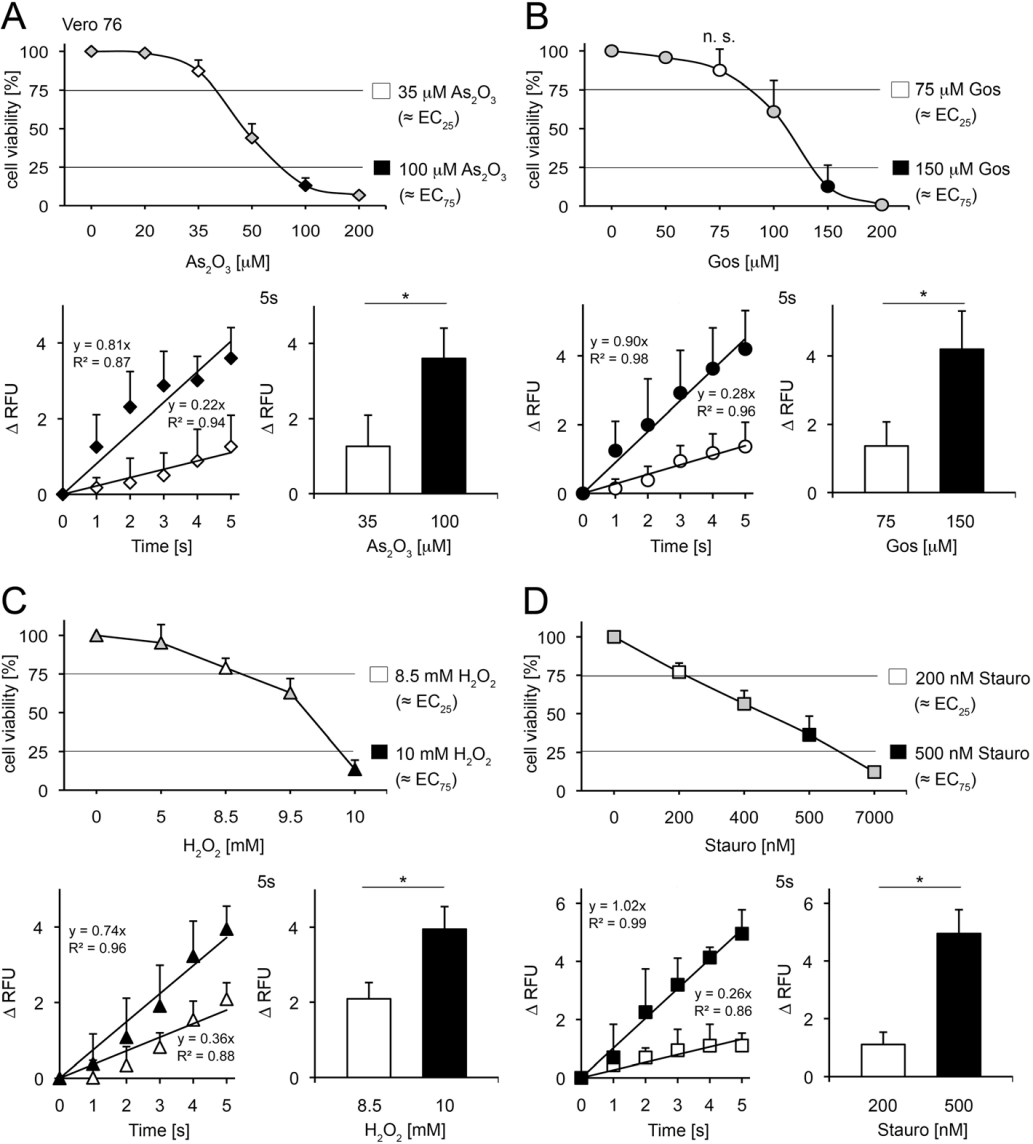
**Assessment of As**_**2**_**O**_**3**_**, gossypol, H**_**2**_**O**_**2 **_**and staurosporine-induced toxicity in Vero 76 cells.** (**A**) Upper panel: Alamar Blue assay in presence of As_2_O_3_ as indicated (mean±SD; n≥3). Lower panel: Fluo-4 analysis of 35 μM and 100 μM As_2_O_3_ treated cells (mean±SD; *p<0.025; n=3; *t* test). (**B**) Upper panel: Alamar Blue assay in presence of gossypol as indicated (mean±SD; n≥3; n.s. not significant; *t* test). Lower panel: Fluo-4 analysis of 75 μm and 150 μM gossypol treated cells (mean±SD; *p<0.025; n=4; *t* test) (**C**) Upper panel: Alamar Blue assay in presence of H_2_O_2_ as indicated (mean±SD; n≥3). Lower panel: Fluo-4 analysis of 8.5 mM and 10 mM H_2_O_2_ treated cells (mean±SD; *p<0.025; n≥3; *t* test) (**D**) Upper panel: Alamar Blue assay in presence of staurosporine as indicated (mean±SD; n≥4). Lower panel: Fluo-4 analysis of 200 nM and 500 nM staurosporine treated cells (mean±SD; *p<0.0025; n=3; *t* test).

Vero 76 cells were analyzed in Fluo-4 assays using the EC_25_ and EC_75_ values for As_2_O_3_, gossypol, H_2_O_2_ and staurosporine as assessed in Alamar Blue assays (Figure 6A-D, Additional file 6A-D). The cytosolic Ca^2+^ level remained robust during the whole analysis period without any toxic challenge (2 h, Additional file 1D).

Sublethal (35 μM) and lethal (100 μM) concentrations of As_2_O_3_ were investigated in Fluo-4 assays (Figure 6A). A dose-dependent linear rise in cytosolic Ca^2+^ was observed within 5 s after toxin treatment (1.26±0.83 RFU versus 3.6±0.81 RFU, Figure 6A). At this time point the cytosolic Ca^2+^ signals showed significant differences between the two doses, which were persistent until 3 h after drug exposure (Additional file 6A).

Gossypol toxicity was investigated at the concentrations of 75 μM and 150 μM in Vero 76 cells (Figure 6B). The increase of cytosolic Ca^2+^ following drug treatment was linear for both concentrations analysed in a dose dependent manner until 5 s post application. The fluorescence units were significantly different between 75 μM and 100 μM gossypol at this time point (1.4±0.71 RFU versus 4.2±1.12 RFU). The difference in rise of cytosolic Ca^2+^ levels seen at 5 s was consistently maintained during the whole period of observation (3 min, 30 min and 3 h, Additional file 6B).

The EC_25_ (8.5 mM) and EC_75_ (10 mM) for H_2_O_2_ in Vero 76 cells as assessed in Alamar Blue viability assays were investigated in Fluo-4 assays (Figure 6C). H_2_O_2_ induced a very fast increase of cytosolic Ca^2+^ at the tested concentrations that was almost linear for the whole time of analysis (30 min, Additional file 6C). The free cytosolic Ca^2+^ elevations of EC_25_ and EC_75_ values were significantly different from control and displayed dose-dependent behaviour already 5 s after drug treatment (Figure 6C).

Comparable results were obtained when Vero 76 cells were challenged with 200 nM or 500 nM staurosporine respectively (Figure 6D, Additional file 6D). Again, as early as 5 s after toxin treatment the cytosolic Ca^2+^ reached significant differences between sublethal (200 nM) and lethal (500 nM) concentrations (1.1±0.43 RFU versus 5.0±0.83 RFU) evident still at 3 h after drug application (Additional file 6D).

### Immediate early drug-induced Ca^2+^ shifts occur independent of the mode of cell death

**Figure 7:**
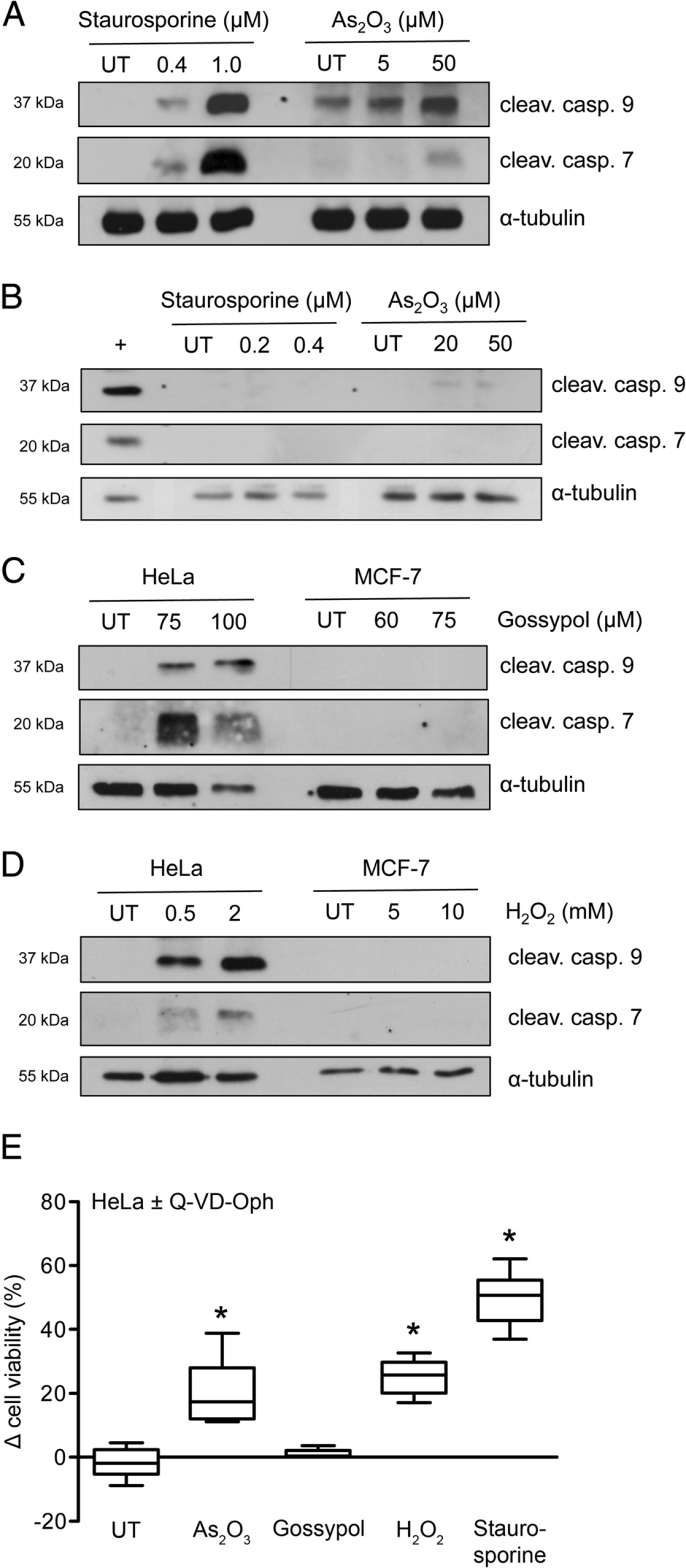
**Caspase activation after sublethal and lethal doses of As**_**2**_**O**_**3**_**, gossypol, H**_**2**_**O**_**2 **_**and staurosporine in HeLa and MCF-7 cells.** (**A**) Western blot analyses of cleaved caspase 7 and 9 after staurosporine and As_2_O_3_ treatment in HeLa cells 4 h post treatment. Α-tubulin is shown as loading control. (**B**) Western blot analyses of cleaved caspase 7 and 9 after staurosporine and As_2_O_3_ treatment in MCF-7 cells 4 h post treatment. Α-tubulin and 1 μM staurosporine treated HeLa cells are shown as controls. (**C**) Western blot analyses of cleaved caspase 7 and 9 after gossypol treatment as indicated in HeLa and MCF-7 cells 4 h post treatment with α-tubulin as loading control. (**D**) Western blot analyses of cleaved caspase 7 and 9 after H_2_O_2_ treatment as indicated in HeLa and MCF-7 cells 4 h post treatment with α-tubulin as loading control. (**E**) Cell viability was assessed with Alamar Blue in HeLa cells in presence or absence of Q-VD-OPh (20 μM). Cells were challenged with EC_75_ values of As_2_O_3_, gossypol, H_2_O_2_ and staurosporine as indicated. Differences in cell survival of Q-VD-OPh plus toxin compared to toxin only treatment are shown (mean±SD; *p<0.05; n=7; *t* test).

We have identified cytosolic Ca^2+^ alterations as an early hallmark of cell death in all tested cell lines, regardless of species origin and of toxin (Figures 3, 4, 5 and 6). Next, we set out to elucidate the mode of cell death in the human cell lines HeLa and MCF-7. When treated with the equitoxic amounts (EC_25_ and EC_75_) of the four test compounds, caspase 7 and 9 were only processed in HeLa cells into their active form as assessed by Western blot analysis 4 h after treatment (Figure 7A-D). By contrast, the cell death in MCF-7 cells was not mediated by activated caspases. The role of caspases in HeLa cells was confirmed by a parallel application of the caspase inhibitor Q-VD-OPh (20 μM) in Alamar Blue viability assays (Figure 7E). Q-VD-OPh could only interfere with As_2_O_3_, H_2_O_2_ and staurosporine-induced cell death, whereas gossypol-mediated viability reduction was not affected by caspase inhibition, despite their activation by all tested toxins and concentration (Figure 7A,C and D).

**Figure 8:**
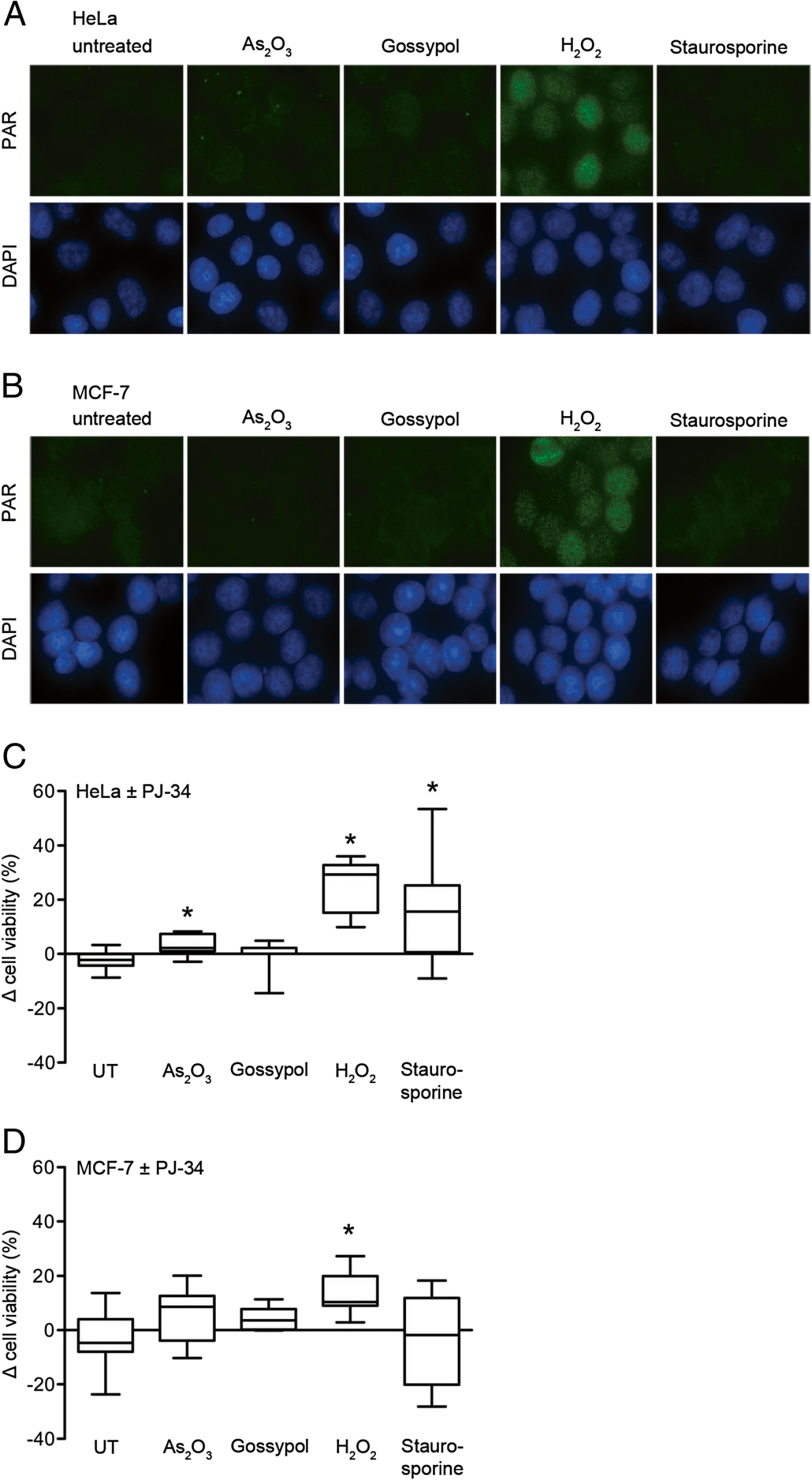
**PAR formation and its effect on cell survival after lethal doses of As**_**2**_**O**_**3**_**, gossypol, H**_**2**_**O**_**2 **_**and staurosporine in HeLa and MCF-7 cells.** (**A**) PAR detection by immunofluorescence in HeLa cells treated with 50 μM As_2_O_3_, 100 μM gossypol, 2 mM H_2_O_2_ or 1 μM staurosporine as described in Methods. Nuclear DAPI staining is shown as control. (**B**) PAR detection by immunofluorescence in MCF-7 cells treated with 50 μM As_2_O_3_, 75 μM gossypol, 10 mM H_2_O_2_ or 0.4 μM staurosporine as described with nuclear DAPI staining as control. (**C**) Cell viability was assessed with Alamar Blue in HeLa cells in presence or absence of PJ-34 (5 μM). Cells were challenged with EC_75_ values of As_2_O_3_, gossypol, H_2_O_2_ and staurosporine as indicated. Differences of cell survival are shown (mean±SD; *p<0.05; n=8; *t* test). (**D**) Cell viability was assessed with Alamar Blue in MCF-7 cells in presence or absence of PJ-34 (5 μM). Cells were challenged with EC_75_ values of As_2_O_3_, gossypol, H_2_O_2_ and staurosporine as indicated. Differences of cell survival are shown (mean±SD; *p<0.05; n=8; *t* test).

Next, we analysed nuclear PARP activity, which is induced immediately after genotoxic insult by binding to strand breaks [46,47]. Subsequent PAR formation accelerates repair processes [47-49], but if PAR is produced in excess due to cytotoxic drug concentrations it also promotes energy collapse, free cytosolic Ca^2+^ overload and the toxic translocation of apoptosis inducing factor (AIF) from mitochondria to the nucleus, leading finally to cell death [19-21,50]. Therefore, nuclear PAR accumulation was investigated 5 min after lethal (EC_75_) challenges with As_2_O_3_, gossypol, H_2_O_2_ and staurosporine in both HeLa (Figure 8A) and MCF-7 cells (Figure 8B). Only the application of EC_75_ levels of H_2_O_2_ but not of As_2_O_3_, gossypol and staurosporine caused detectable levels of nuclear PAR 5 min after treatment in immunofluorescence microscopy experiments. Interestingly, in HeLa cells the PARP inhibitor PJ-34 could not only interfere with H_2_O_2_-, but also with As_2_O_3_- and staurosporine-induced cell death (Figure 8C), pointing to PARP activity as a common feature in these different cell killing agents, even if PAR levels are below detection limit. By contrast, the application of PJ-34 was exclusively protective in H_2_O_2_-induced loss of viability in MCF-7 cells (Figure 8D). Gossypol-induced cell death was not affected by PARP inhibition in both tested cell lines.

## Discussion

The development of drugs and chemicals requires extensive cytotoxicity testing. Several tests rely on the energy status and the oxidative capacity of cells, i.e. the MTT and the Alamar Blue assay [3]. Both can be applied in an automated way on multi-well plates for HTS. But there are certain limitations, as the final readout depends on two incubation steps: the exposure to the substance and the biotransformation of the reagent. Additionally, the cost effectiveness is a serious factor in large scale screening.

In recent publications, we reported a correlation between cytosolic Ca^2+^ increase and cell death induced by oxidative stress [20,21]. Using a panel of different biological and pharmacological approaches we investigated distinct Ca^2+^ sources merging in a composite pool of toxin dependent increase in free cytosolic Ca^2+^. The enzymatic activities of the nuclear PARP1 in conjunction with its counterpart poly(ADP-ribose) glycohydrolase (PARG) are responsible for extracellular Ca^2+^ gated by transmembranous transient receptor mediated Ca^2+^ channel (TRPM2). On the other hand, free cytosolic Ca^2+^ origins also from intracellular sources. For instance, protein markers of endoplasmic reticulum (ER) stress were detected pointing to Ca^2+^ released from ER stores in parallel. Blocking the influx of Ca^2+^ protected the cells from oxidative insults.

In order to see whether Ca^2+^ shifts are generally predictive of cytotoxicity, we investigated here a wide spectrum of toxins in cell lines from different species origin. The toxicity of arsenic trioxide, hydrogen peroxide, gossypol and staurosporine was tested in human, mouse, and monkey cells using Alamar Blue assay. These compounds have different cellular targets and induce different cell death pathways, ranging from general macromolecule damage, especially to DNA, by oxidative stressor H_2_O_2_ to the apoptotic model compound staurosporine, which has been shown to inhibit a wide spectrum of kinases without damaging DNA. The toxicity data were compared to cytosolic Ca^2+^ measurements at the respective sublethal (EC_25_) and lethal (EC_75_) doses. Our fluorimetric assay revealed in all settings a rapid rise in cytosolic Ca^2+^, regardless of species-origin and toxin applied. Moreover, it has a low LOD. Thus, our data provide evidence that Ca^2+^ shifts are a common denominator in cytotoxic insults, independent of the mode of cell death. Interestingly, this can be monitored with an unmatched speed and at doses that show hardly significant changes in cell viability assays. Even sublethal (EC_25_) toxin concentrations generated slopes of free cytosolic Ca^2+^ increases significantly different from solvent controls indicative for the superior sensitivity of the Fluo-4 Ca^2+^ assay. Moreover, this assay discriminates between structurally closely related titanium(IV)-salane complexes, i.e. toxic TC52 and non-toxic TC53. In an additional data set, we tested the toxicity of a physiological compound, i.e. ATP. High extracellular concentrations have been reported to induce cell death [35,39-45]. Indeed, we also detected free cytosolic Ca^2+^ shifts in our assay after application of ATP in a similar setting as before (EC_25_ and EC_75_). However, low dose extracellular ATP induces Ca^2+^ shifts if cells express members of P2X and P2Y transporter family, as it is the case in HeLa cells [38]. Therefore, in this specific cell line and setting, we cannot rule out the occurrence of false-positives. Falsely categorizing a substance as positive or negative due to specific characteristics of the tested cells is always a risk in cytotoxicity screens. For example bleomycin, a well-established clastogenic agent and anti-tumor drug has to be taken up via the hCT2-transporter, which is the rate-limiting step determining its toxic activity as reviewed recently [51]. To avoid false-negative and false-positive results we suggest testing a panel of cell lines, which differ in their receptor repertoire. It can be expected that physiological molecules will obviously induce cellular responses including Ca^2+^ dependent signaling processes. In contrast, engineered substances inducing a rise in free cytosolic Ca^2+^ as presented in this study are indicative of unwanted biological effects. Therefore we conclude that cytosolic Ca^2+^ increases within the first 5 s of exposure as measured with Fluo-4 dye are predictive of the cytotoxic potential of a xenobiotic compound.

## Conclusions

Our newly developed assay is applicable in cells from different species and with a wide variety of toxins, acting on different signaling pathways and modes of cell death. Measuring the free cytosolic Ca^2+^ increase in the first 5 s of exposure shows the same or even higher statistical predictivity than the standard Alamar Blue assay. Thus, this fluorimetry-based method is a rapid predictor of cytotoxicity, superior to other assays in speed and cost effectiveness.

## Methods

### Cell culture

In this study HeLa, immortalized mouse embryonic fibroblasts, MCF-7 and Vero 76 cells were investigated (Figure 2B). All cell monolayers were cultured at 37°C in a water-saturated (5% CO_2_) atmosphere, in complete Dulbecco’s modified Eagle’s medium (D-MEM, Gibco, Lucerne, Switzerland) containing 1 g/L glucose and supplemented with 10% (v/v) FBS and Penicillin/Streptomycin (Invitrogen, Lucerne, Switzerland).

### Chemicals

*N*-(2-Quinolyl)valyl-aspartyl-(2,6-difluorophenoxy)methylketone (Q-VD-OPh) was from Calbiochem (Zug, Switzerland). *N*-(6-Oxo-5,6-dihydro-phenanthridin-2-yl)-*N,N*-dimethylacetamide, HCl (PJ-34) was obtained from ENZO Life Sciences (Lausen, Switzerland). HOECHST 33342 was from Invitrogen. Titanium(IV)-salane complexes TC52 and TC53 were both synthesized in the Chemistry Department (Thomas Huhn Group) of University of Konstanz/Germany. All other chemicals were from Applichem (Baden-Dättwil, Switzerland), Fluka (Buchs, Switzerland), Merck (Zug, Switzerland) or Sigma. All chemicals used as inhibitors were simultaneously administered with toxin treatment.

### Toxin treatment

Cells were challenged with 1 part (50 μL) H_2_O_2_ (Sigma, Buchs, Switzerland) diluted in OPTI-MEM I (Gibco) to the desired concentration. After 1 h, 3 parts (150 μL) complete D-MEM were added. Gossypol (Sigma) was dissolved in DMSO to a stock solution of 100 mM. Then diluted in OPTI-MEM I to the desired concentration. Staurosporine (Sigma, dissolved in DMSO to a stock solution of 1 mM) and As_2_O_3_ (Sigma, dissolved in H_2_O alkalized with NaOH to a stock solution of 5 mM) were diluted in D-MEM directly to the concentration needed. TC52 and TC53 were dissolved in DMSO to a stock solution of 2.5 mM and diluted in D-MEM to the desired concentration. ATP Mg^2+^ salt (Sigma) was diluted in PBS supplemented with 2 mM Ca^2+^ to the concentration needed. After 30 min of treatment the ATP solution was replaced with complete D-MEM. All toxin treatments were maintained without any alterations until the end of the experiment.

### Alamar blue viability assay

Cells were seeded in 96-well-plates (15 000 cells/well) and incubated overnight (Figure 1C). Cells were treated with the toxins as described above. After 20 h (with TC52 and TC53 treatment 44 h), medium was replaced with 200 μL D-MEM 10% (v/v) Alamar Blue (Biozol, Eching, Germany). After 3 or 4 h, fluorescence was monitored at wavelength 530 nm for excitation and 590 nm for emission in LS55 luminescence spectrometer (Perkin-Elmer, Schwerzenbach, Switzerland).

### Calcium measurements

This was performed as described before [20]. Briefly, 20 000 cells/well in 96-well-plates (Costar Corning Incorporated, Baar, Switzerland) were washed twice with 49 parts of calcium-free HBSS (0.49 mM MgCl_2_, 0.41 mM MgSO_4_, 5.33 mM KCl, 0.44 mM KH_2_PO_4_, 4.17 mM NaHCO_3_, 137 mM NaCl, 0.34 mM Na_2_HPO_4_, 5.56 mM Dextrose) supplemented with 1 part 1 M HEPES (pH 7.2) (Assay Buffer) containing CaCl_2_ or not. 100 μL Fluo-4-NW-dye-mix from Molecular Probes (Invitrogen) was added and incubated for 30 min at 37°C, followed by 30 min incubation in the dark at room temperature (Figure 1C). Changes in relative fluorescence units (ΔRFU) from the Fluo-4-NW-dye quantify alterations in free cytosolic Ca^2+^ concentrations (excitation/emission 485/535 nm; slits 10/15 nm) in LS55 luminescence spectrometer (Perkin-Elmer) after toxin treatment. Stock solutions of toxins were diluted in Assay Buffer to the desired concentration. Free cytosolic Ca^2+^ was monitored for the indicated time with a measure frequency of 1 s or less.

### Western blot detection

Immunoblots were performed as described previously [20]. The following primary antibodies were used: anti-cleaved-caspase-7 (Asp198, Cell Signaling; 1:1 000), anti-cleaved-caspase-9 (Asp315, Cell Signaling; 1:1 000) anti-α-Tubulin (Cell Signaling; 1:5 000). All secondary antibodies were from Sigma. Equal quantities of protein were loaded into each lane for SDS-PAGE separation as controlled by the simultaneous use of α-Tubulin as internal protein standards.

### Immunofluorescence of PAR

Cells were seeded on coverslips (Thermo Scientific, Allschwil, Switzerland) in 24-well-plates (Costar Corning Incorporated) and let attach overnight. The toxin treatment was performed in D-MEM for 5 min. Cells were fixed with ice-cold methanol and stored at −20°C for 7 min. Coverslips were subsequently washed twice with 1xTris buffered saline (TBS, pH 7.4, 3 min at room temperature) and incubated with Blocking Buffer (1xTBS/0.2% Tween 20 (TBST), 1% BSA) for 30 min at 37°C. Monoclonal 10H anti-poly(ADP-ribose) (PAR) antibody [52] was used as 1^st^ antibody (diluted 1:200 in Blocking Buffer). After an incubation for 1 h at 37°C, coverslips were washed three times with TBST (each 5 min), followed by a 2^nd^ antibody incubation (Alexa Fluor 488-conjugated, 1:200 in blocking solution) for 1 h at 37°C in the dark. Afterwards, probes were washed three times with TBST (each 5 min). DAPI staining was performed for 5 min and coverslips were washed with H_2_O and dried afterwards. The samples were further processed with ProLong Antifade kit (Invitrogen) according to the manufacturer’s protocol and analyzed with a fluorescence microscope (Nikon) connected to a digital camera (Kappa, Grenchen, Switzerland).

### Statistical analysis

If not stated differently, all results are shown as mean±SD of the indicated number of independent experiments. All statistical analyses were calculated with Prism Software 5.0b (GraphPad Software, San Diego California USA).

## Competing interests

The authors declare that they have competing interests. A patent application protecting the invention has been filed (EP 12/187234).

## Authors’ contribution

PW, CB and TP planned and performed the experiments. All authors analysed the data. PW, CB, SB and FRA wrote the manuscript. All authors read and approved the final manuscript.

## Supplementary Material

Additional file 1 **Control measurements of Fluo-4 free cytosolic calcium assay.** (**A**) HeLa cells (mean±SD, n=2). (**B**) MCF-7 cells (mean±S.D., n=2). (**C**) Murine fibroblasts (mean±SD, n=2). (**D**) Vero 76 cells (mean±SD, n=3). (**E**) Ca^2+^ shift endpoint at 5 s after 1 μM, 2 μM or 5 μM As_2_O_3_ with (mean±SD; n≥3; *t* test) compared to control in HeLa cells. Ca^2+^ shift endpoint at 5 s after 5 μM, 10 μM or 75 μM gossypol with (mean±SD; n≥4; *t* test) compared to control. Ca^2+^ shift endpoint at 5 s after 100 nM, 200 nM or 400 nM staurosporine with (mean±SD; n≥3; *t* test) compared to control. (**F**) Alamar Blue endpoint at 24 h after 1 μM, 2 μM or 5 μM As_2_O_3_ with (mean±SD; n≥3; *t* test) compared to control in HeLa cells. Alamar Blue endpoint at 24 h after 5 μM, 10 μM or 75 μM gossypol with (mean±SD; n≥3; *t* test) compared to control. Alamar Blue endpoint at 24 h after 100 nM, 200 nM or 400 nM staurosporine with (mean±SD; n≥3; *t* test) compared to control. (**G**) Chemical structures of the investigated compounds TC52 and TC53. (**H**) Alamar Blue endpoint at 24 h after 4 μM or 10 μM TC52 or 10 μM TC53 with (mean±SD; n=3; *t* test) compared to control in HeLa cells (**I**) Ca^2+^ shift endpoint at 5 s after 4 μM or 10 μM TC52 or 10 μM TC53 with (mean±SD; n=3; *t* test) compared to untreated control in HeLa cells. Click here for file

Additional file 2 **Impact of toxic compounds on cytosolic Ca**^**2+ **^**levels in HeLa cells.** (**A**) Ca^2+^ shifts after 5 μM or 50 μM As_2_O_3_ with (mean±SD; *p<0.0005; n≥3; *t* test) at 1800 s. (**B**) Ca^2+^ shifts after 75 μM or 100 μM gossypol with (mean±SD; *p<0.0025; n=3; *t* test) at 1800 s. (**C**) Ca^2+^ shifts after 0.5 mM or 2 mM H_2_O_2_ with (mean±SD; *p<0.001; n≥4; *t* test) at 180 s and (mean±SD; *p<0.001; n≥4; *t* test) at 1800 s. (**D**) Ca^2+^ shifts after 400 nM or 1000 nM staurosporine with (mean±SD; *p<0.025; n=3; *t* test) at 1800 s. Click here for file

Additional file 3 **Assessment of ATP-induced toxicity in HeLa cells.** (**A**) Alamar Blue assay in presence of ATP as indicated (mean±SD; n≥4). (**B**) Ca^2+^ shifts after 25 mM or 40 mM ATP (mean±SD; n≥7). (**C**) Statistical evaluation of 25 or 40 mM ATP treated HeLa cells in Fluo-4 analyses (mean±SD; *p<0.0005; n=7; *t* test). Click here for file

Additional file 4 **Impact of toxic compounds on cytosolic Ca**^**2+ **^**levels in MCF-7 cells.** (**A**) Ca^2+^ shifts after 20 μM or 50 μM As_2_O_3_ with (mean±SD; *p<0.01; n=3; *t* test) at 10800 s. (**B**) Ca^2+^ shifts after 60 μM or 75 μM gossypol with (mean±SD; *p<0.001; n=3; *t* test) at 1800 s. (**C**) Ca^2+^ shifts after 5 mM or 10 mM H_2_O_2_ with (mean±SD; *p=0.0001; n≥3; *t* test) at 1800 s. (**D**) Ca^2+^ shifts after 200 nM or 400 nM staurosporine with (mean±SD; *p<0.01; n=3; *t* test) at 180 s and (mean±SD; *p<0.005; n=3; *t* test) at 1800 s and (mean±SD; *p<0.0025; n=3; *t* test) at 10800 s. Click here for file

Additional file 5 **Impact of toxic compounds on cytosolic Ca**^**2+ **^**levels in murine fibroblasts.** (**A**) Ca^2+^ shifts after 45 μM or 50 μM As_2_O_3_ with (mean±SD; *p<0.025; n=4; *t* test) at 180 s. (**B**) Ca^2+^ shifts after 75 μM or 100 μM gossypol with (mean±SD; *p<0.025; n=3; *t* test) at 180 s and (mean±SD; *p=0.0001; n=3; *t* test) at 1800 s. (**C**) Ca^2+^ shifts after 0.5 mM or 5 mM H_2_O_2_ with (mean±SD; *p<0.005; n≥3; *t* test) at 180 s and (mean±SD; *p<0.01; n≥3; *t* test) at 1800 s. (**D**) Ca^2+^ shifts after 500 nM or 4000 nM staurosporine with (mean±SD; not significant; n=3; *t* test) at 180 and 1800 s. Click here for file

Additional file 6 **Impact of toxic compounds on cytosolic Ca**^**2+ **^**levels in Vero 76 cells.** (**A**) Ca^2+^ shifts after 35 μM or 100 μM As_2_O_3_ with (mean±SD; *p<0.0025; n≥3; *t* test) at 10800 s. (**B**) Ca^2+^ shifts after 75 μM or 150 μM gossypol with (mean±SD; *p<0.025; n≥3; *t* test) at 180 s with (mean±SD; *p<0.005; n=3; *t* test) at 1800 s and (mean±SD; *p<0.0001; n≥3; *t* test) at 10800 s. (**C**) Ca^2+^ shifts after 8.5 mM or 10 mM H_2_O_2_ with (mean±SD; *p<0.005; n=3; *t* test) at 1800 s. (**D**) Ca^2+^ shifts after 200 nM or 500 nM staurosporine with (mean±SD; *p<0.05; n=3; *t* test) at 10800 s. Click here for file
