# Supplementary figures and images for: Cytosolic Ca2+ shifts as early markers of cytotoxicity
Source: Cell Commun Signal. 2013 Feb 6;11:11. doi: 10.1186/1478-811X-11-11 (PMC3762065; doi:10.1186/1478-811X-11-11)

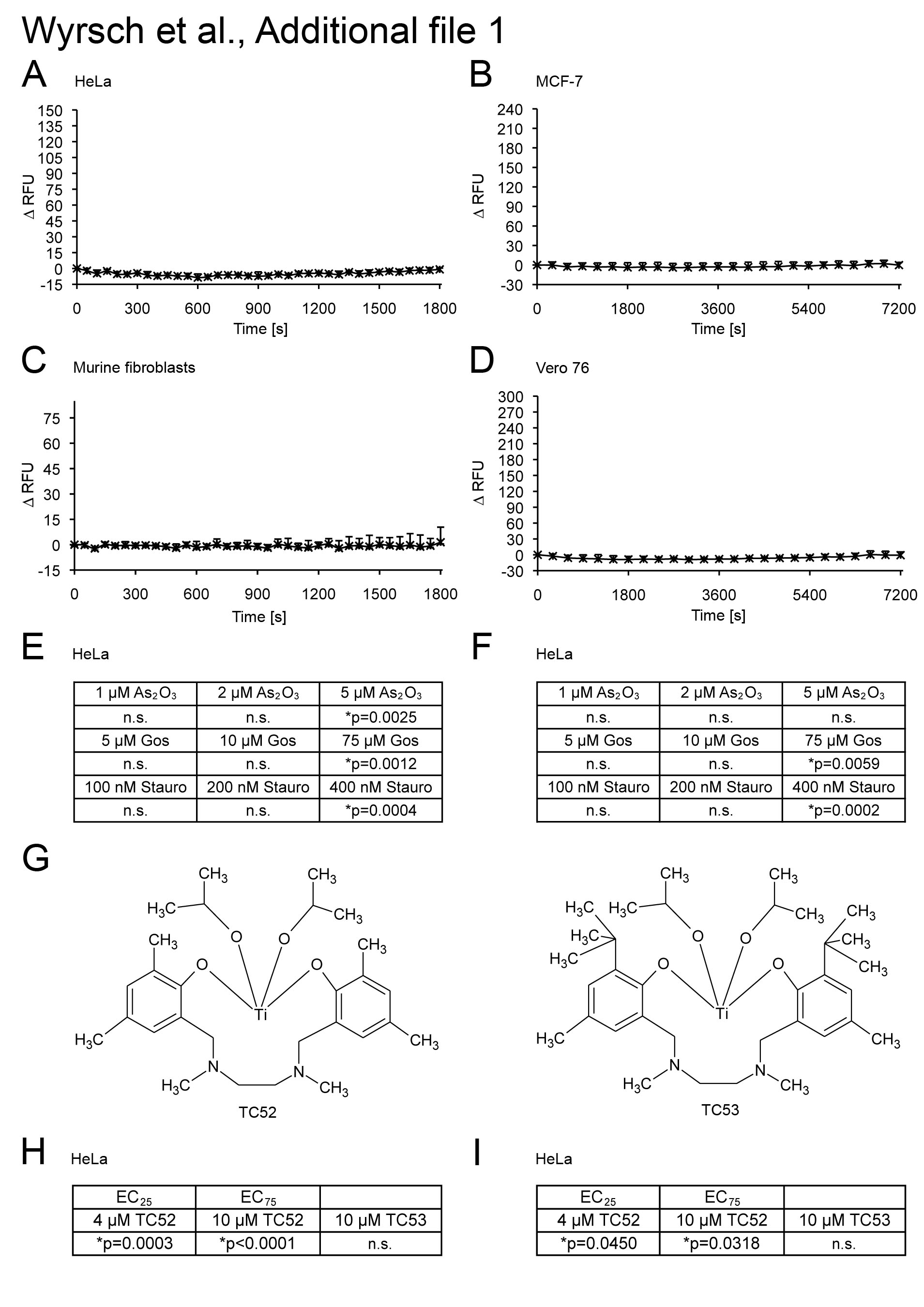

Supplement: Additional file 1 — Control measurements of Fluo-4 free cytosolic calcium assay. (A) HeLa cells (mean±SD, n=2). (B) MCF-7 cells (mean±S.D., n=2). (C) Murine fibroblasts (mean±SD, n=2). (D) Vero 76 cells (mean±SD, n=3). (E) Ca2+ shift endpoint at 5 s after 1 μM, 2 μM or 5 μM As2O3 with (mean±SD; n≥3; t test) compared to control in HeLa cells. Ca2+ shift endpoint at 5 s after 5 μM, 10 μM or 75 μM gossypol with (mean±SD; n≥4; t test) compared to control. Ca2+ shift endpoint at 5 s after 100 nM, 200 nM or 400 nM staurosporine with (mean±SD; n≥3; t test) compared to control. (F) Alamar Blue endpoint at 24 h after 1 μM, 2 μM or 5 μM As2O3 with (mean±SD; n≥3; t test) compared to control in HeLa cells. Alamar Blue endpoint at 24 h after 5 μM, 10 μM or 75 μM gossypol with (mean±SD; n≥3; t test) compared to control. Alamar Blue endpoint at 24 h after 100 nM, 200 nM or 400 nM staurosporine with (mean±SD; n≥3; t test) compared to control. (G) Chemical structures of the investigated compounds TC52 and TC53. (H) Alamar Blue endpoint at 24 h after 4 μM or 10 μM TC52 or 10 μM TC53 with (mean±SD; n=3; t test) compared to control in HeLa cells (I) Ca2+ shift endpoint at 5 s after 4 μM or 10 μM TC52 or 10 μM TC53 with (mean±SD; n=3; t test) compared to untreated control in HeLa cells. [file 1478-811X-11-11-S1.jpeg]

# Wyrsh et al., Additional file 2

A

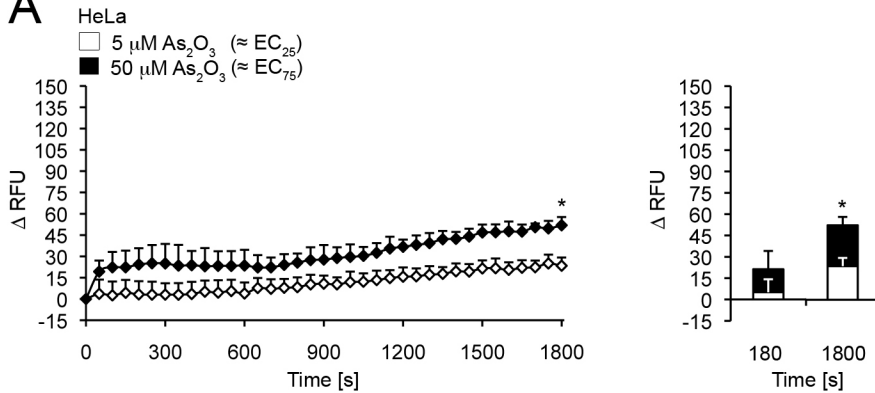

B

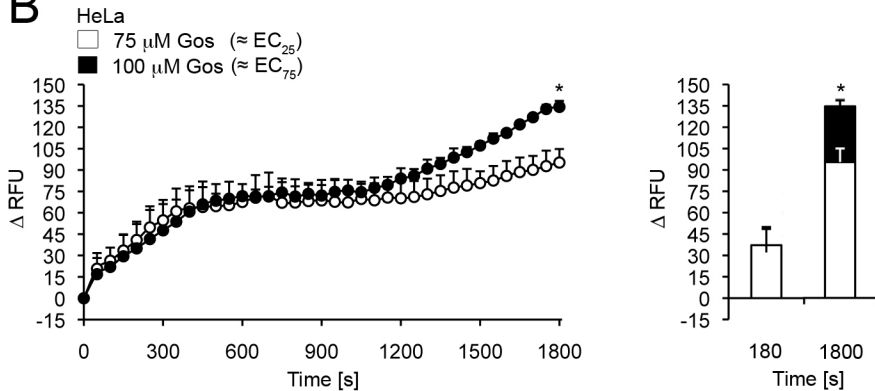

C

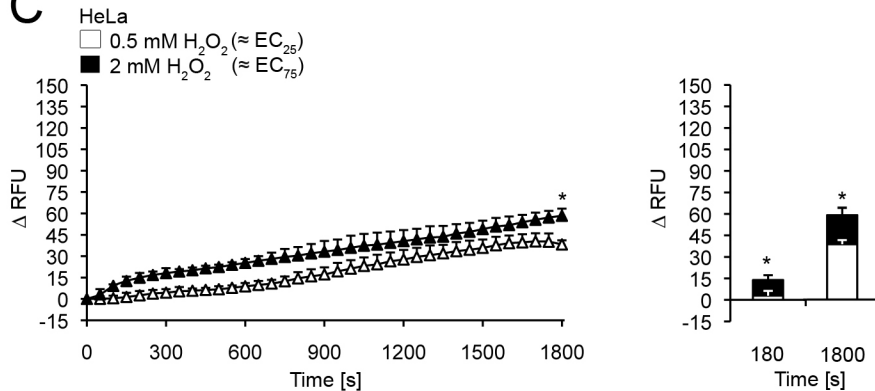

D

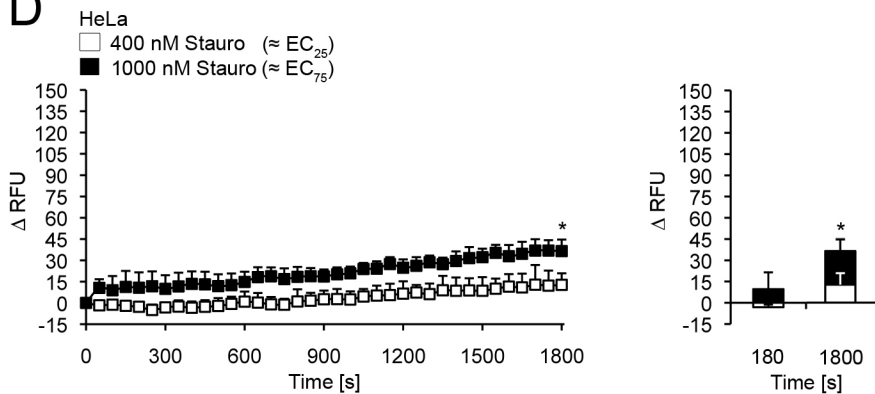

Supplement: Additional file 2 — Impact of toxic compounds on cytosolic Ca2+ levels in HeLa cells. (A) Ca2+ shifts after 5 μM or 50 μM As2O3 with (mean±SD; *p<0.0005; n≥3; t test) at 1800 s. (B) Ca2+ shifts after 75 μM or 100 μM gossypol with (mean±SD; *p<0.0025; n=3; t test) at 1800 s. (C) Ca2+ shifts after 0.5 mM or 2 mM H2O2 with (mean±SD; *p<0.001; n≥4; t test) at 180 s and (mean±SD; *p<0.001; n≥4; t test) at 1800 s. (D) Ca2+ shifts after 400 nM or 1000 nM staurosporine with (mean±SD; *p<0.025; n=3; t test) at 1800 s. [file 1478-811X-11-11-S2.pdf]

# Wyrsh et al., Additional file 3

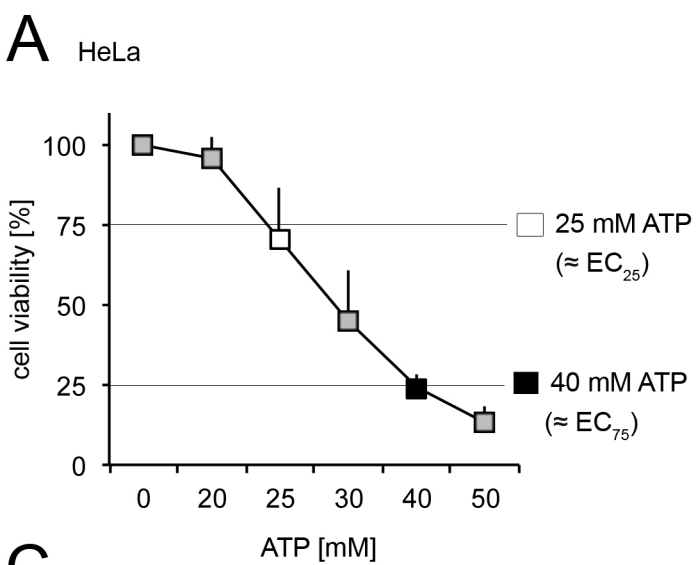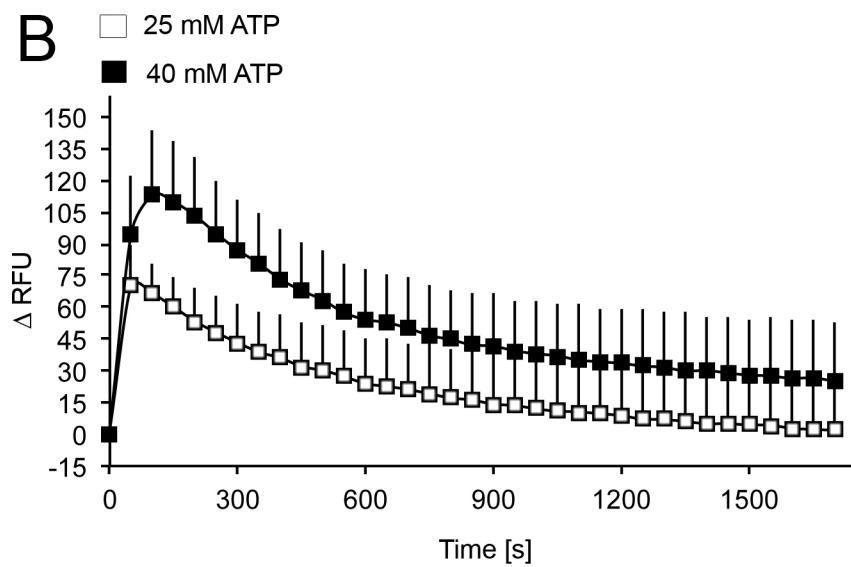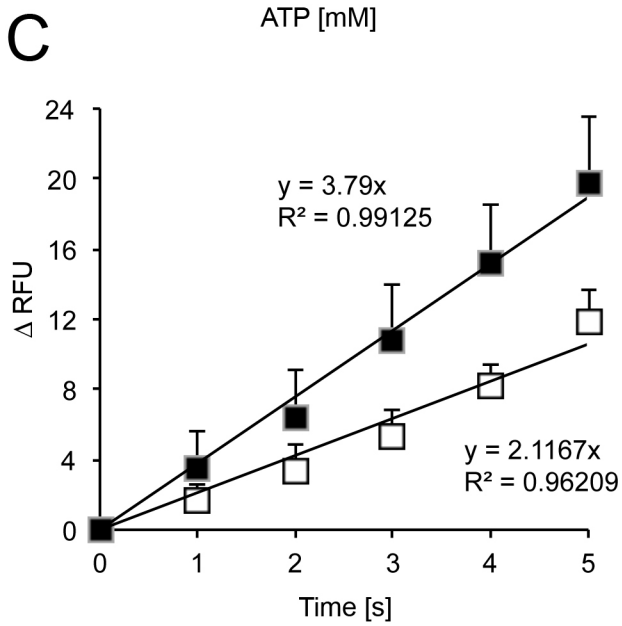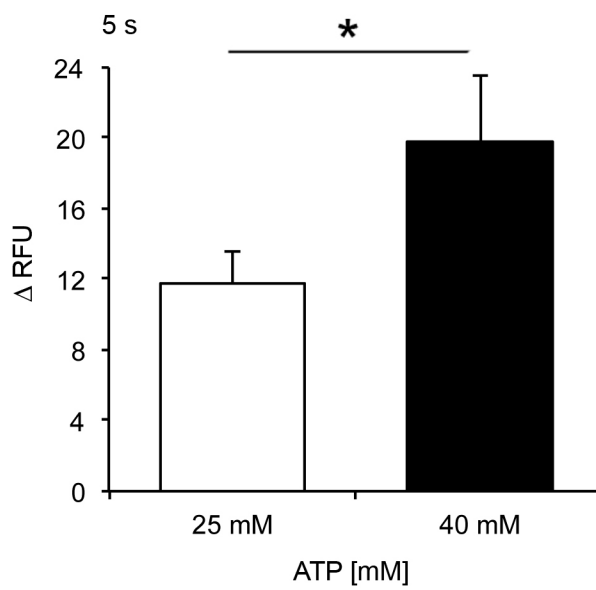

Supplement: Additional file 3 — Assessment of ATP-induced toxicity in HeLa cells. (A) Alamar Blue assay in presence of ATP as indicated (mean±SD; n≥4). (B) Ca2+ shifts after 25 mM or 40 mM ATP (mean±SD; n≥7). (C) Statistical evaluation of 25 or 40 mM ATP treated HeLa cells in Fluo-4 analyses (mean±SD; *p<0.0005; n=7; t test). [file 1478-811X-11-11-S3.pdf]

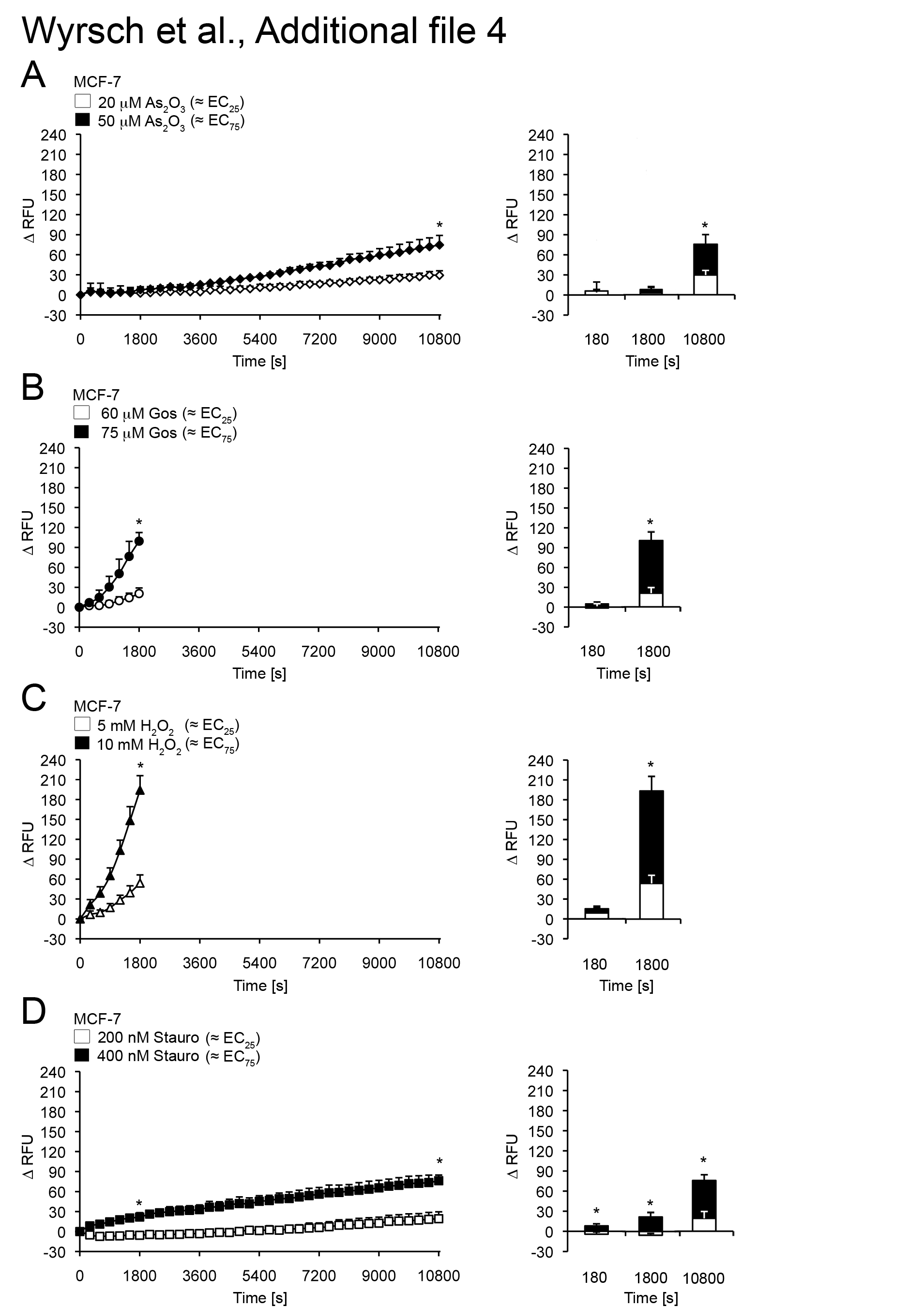

Supplement: Additional file 4 — Impact of toxic compounds on cytosolic Ca2+ levels in MCF-7 cells. (A) Ca2+ shifts after 20 μM or 50 μM As2O3 with (mean±SD; *p<0.01; n=3; t test) at 10800 s. (B) Ca2+ shifts after 60 μM or 75 μM gossypol with (mean±SD; *p<0.001; n=3; t test) at 1800 s. (C) Ca2+ shifts after 5 mM or 10 mM H2O2 with (mean±SD; *p=0.0001; n≥3; t test) at 1800 s. (D) Ca2+ shifts after 200 nM or 400 nM staurosporine with (mean±SD; *p<0.01; n=3; t test) at 180 s and (mean±SD; *p<0.005; n=3; t test) at 1800 s and (mean±SD; *p<0.0025; n=3; t test) at 10800 s. [file 1478-811X-11-11-S4.jpeg]

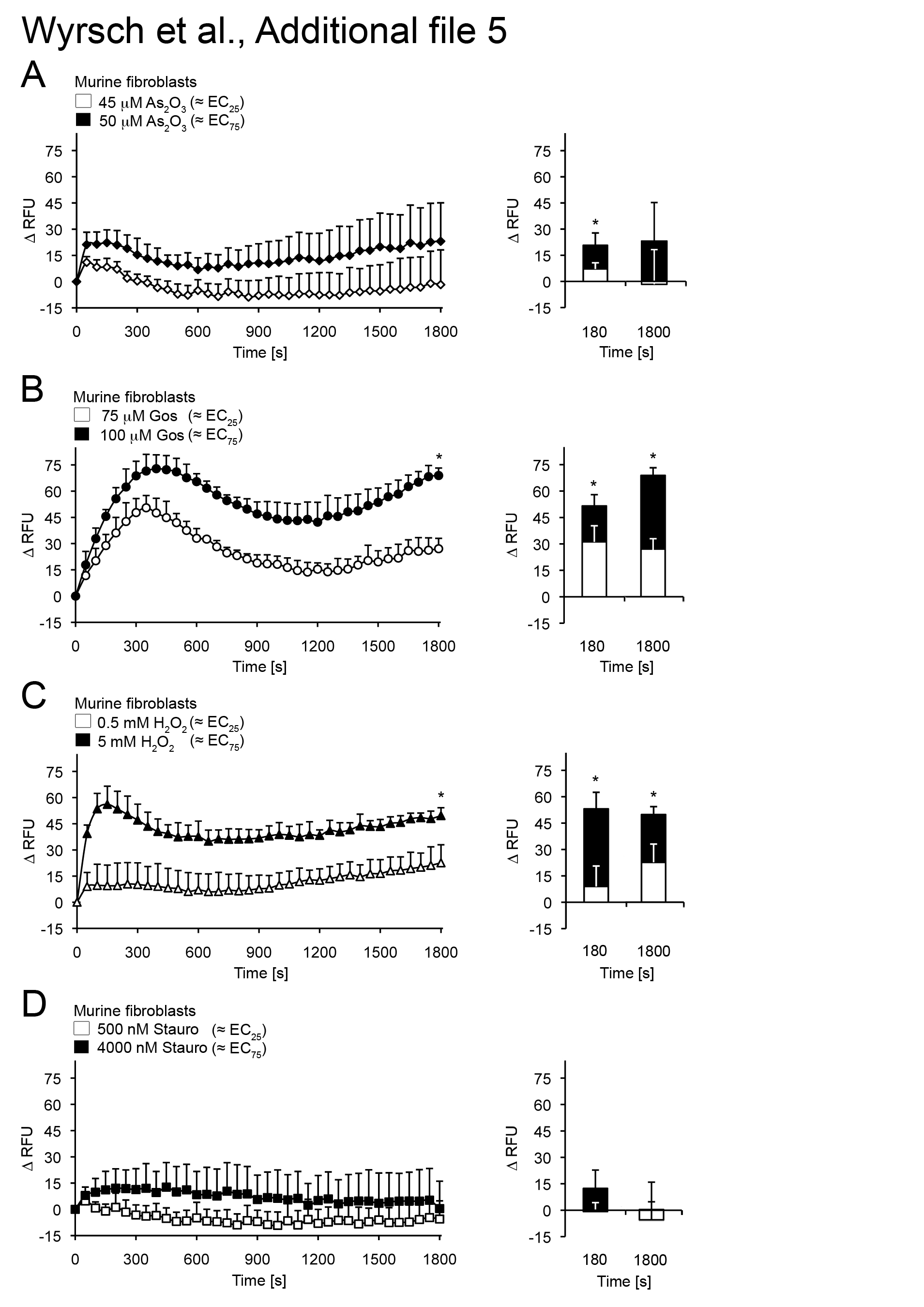

Supplement: Additional file 5 — Impact of toxic compounds on cytosolic Ca2+ levels in murine fibroblasts. (A) Ca2+ shifts after 45 μM or 50 μM As2O3 with (mean±SD; *p<0.025; n=4; t test) at 180 s. (B) Ca2+ shifts after 75 μM or 100 μM gossypol with (mean±SD; *p<0.025; n=3; t test) at 180 s and (mean±SD; *p=0.0001; n=3; t test) at 1800 s. (C) Ca2+ shifts after 0.5 mM or 5 mM H2O2 with (mean±SD; *p<0.005; n≥3; t test) at 180 s and (mean±SD; *p<0.01; n≥3; t test) at 1800 s. (D) Ca2+ shifts after 500 nM or 4000 nM staurosporine with (mean±SD; not significant; n=3; t test) at 180 and 1800 s. [file 1478-811X-11-11-S5.jpeg]

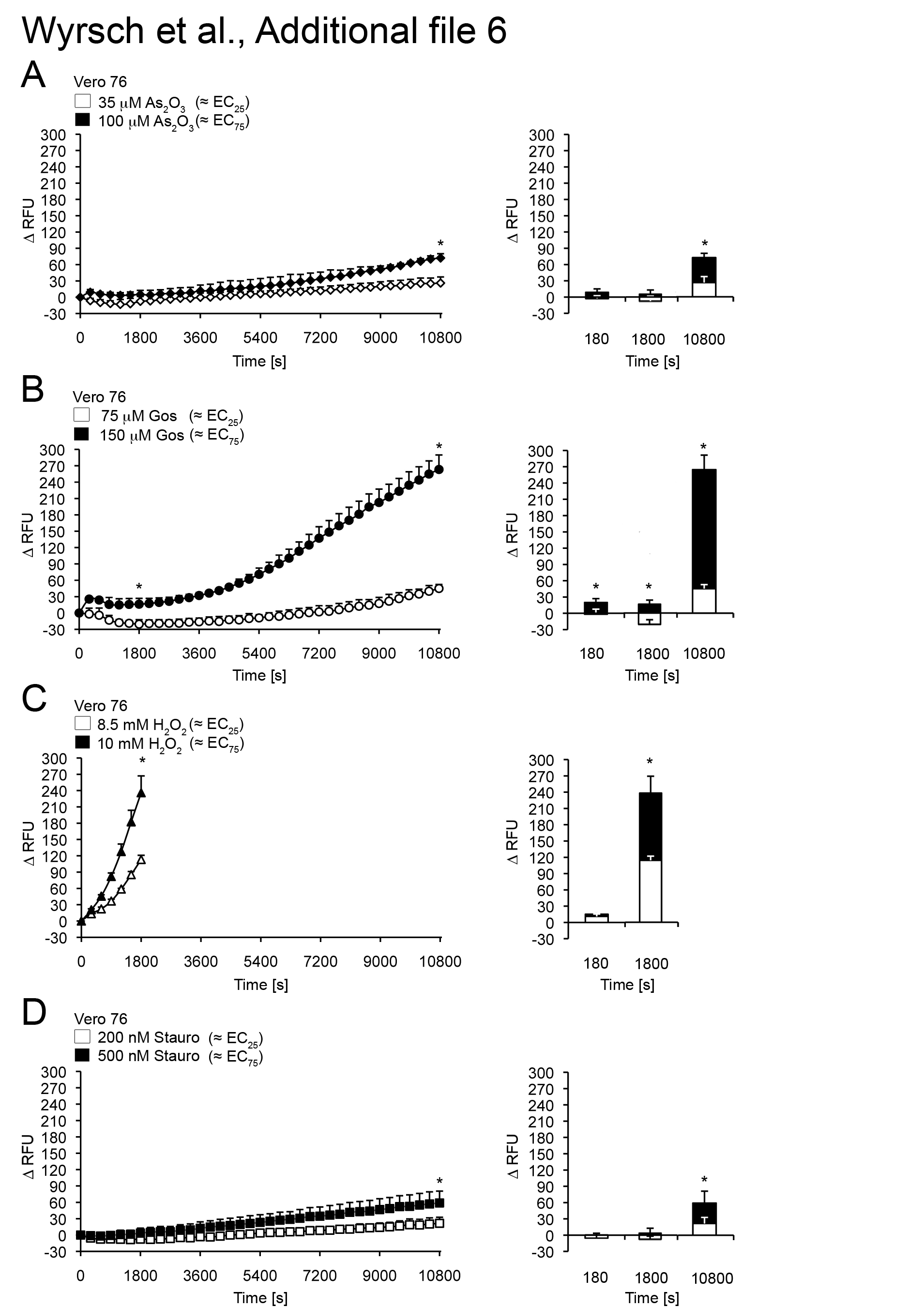

Supplement: Additional file 6 — Impact of toxic compounds on cytosolic Ca2+ levels in Vero 76 cells. (A) Ca2+ shifts after 35 μM or 100 μM As2O3 with (mean±SD; *p<0.0025; n≥3; t test) at 10800 s. (B) Ca2+ shifts after 75 μM or 150 μM gossypol with (mean±SD; *p<0.025; n≥3; t test) at 180 s with (mean±SD; *p<0.005; n=3; t test) at 1800 s and (mean±SD; *p<0.0001; n≥3; t test) at 10800 s. (C) Ca2+ shifts after 8.5 mM or 10 mM H2O2 with (mean±SD; *p<0.005; n=3; t test) at 1800 s. (D) Ca2+ shifts after 200 nM or 500 nM staurosporine with (mean±SD; *p<0.05; n=3; t test) at 10800 s. [file 1478-811X-11-11-S6.jpeg]
